# Insights into the Antimicrobial Mechanisms of a Scorpion Defensin on *Staphylococcus aureus* Using Transcriptomic and Proteomic Analyses

**DOI:** 10.3390/molecules30071542

**Published:** 2025-03-30

**Authors:** Xuhua Yang, Haozhen Zhang, Sijia Lu, Yiyuan Guo, Yitong Li, Chenhu Qin, Zheng Zuo, Yingliang Wu, Zhijian Cao

**Affiliations:** 1 National “111” Center for Cellular Regulation and Molecular Pharmaceutics, Key Laboratory of Fermentation Engineering (Ministry of Education), Hubei University of Technology, Wuhan 430068, China; yangxh@whu.edu.cn; 2 College of Life Sciences, Wuhan University, Wuhan 430072, China; zhanghaozhen@whu.edu.cn (H.Z.); 2022202040009@whu.edu.cn (S.L.); 2019202040010@whu.edu.cn (Y.G.); 2023202040154@whu.edu.cn (Y.L.); 2014301060068@whu.edu.cn (Z.Z.); 3 Department of Biochemistry and Molecular Biology, College of Basic Medicine, Hubei University of Medicine, Shiyan 442000, China; 2016202040025@whu.edu.cn

**Keywords:** transcriptome, proteome, defensin, BmKDfsin4, bacterium, *Staphylococcus aureus*, antibacterial mechanism

## Abstract

Defensins constitute a family of cationic antimicrobial peptides that act against different bacteria; however, global information regarding their antibacterial mechanisms from omics-based analyses is highly limited. In this study, transcriptomics and proteomics were used to explore the antibacterial mechanisms of defensin (BmKDfsin4) originally isolated from a scorpion on a common Gram-positive bacterium. *Staphylococcus aureus* (AB94004) was treated with BmKDfsin4 for 15, 30, or 45 min based on its ability to moderately inhibit bacterial growth for one hour. Compared with those in the control group, more than 1000 genes and nearly 500 proteins in *S. aureus* were significantly differentially expressed after BmKDfsin4 treatment. In-depth analysis revealed that BmKDfsin4 significantly upregulated bacterial ribosome-related pathways and ribosomal components. In contrast, BmKDfsin4 also significantly downregulated the synthesis and metabolism pathways of bacterial amino acids. Moreover, BmKDfsin4 inhibited the synthesis pathways of teichoic acid and peptidoglycan, which are the key components of the cell wall in *S. aureus*. Furthermore, glycolysis and other metabolic processes in *S. aureus* were markedly reduced by BmKDfsin4. Overall, the global information detected from *S. aureus* revealed the multiple antibacterial mechanisms of BmKDfsin4, which could encourage the exploration of global bacterial information from the defensin family with high degrees of sequence variability and accelerate the research and development of defensins as new antibacterial agents.

## 1. Introduction

Bacteria represent one of the major threats to human health, and severe bacterial infections can even lead to death [1,2,3]. Due to the increasing risk of antibiotic resistance in bacteria, extensive investigations conducted in recent years have focused on the antibacterial functions and mechanisms of small molecules, linear antimicrobial peptides, and cysteine-rich defensins [4,5,6,7,8]. Currently, the global differential expression profiles of bacterial genes and proteins are enriching the antibacterial knowledge of antibacterial reagents based on transcriptomic and proteomic analyses, which can accelerate the research and development of novel antibacterial drugs [9,10].

Many omics-based analyses have indicated that small molecules generally target bacterial cell membranes or cell walls. The traditional use of vancomycin has also been found to target specific stages of bacterial cell wall synthesis based on proteomic analysis [11]. In contrast to the antibacterial mechanism of traditionally used vancomycin, proteomic analysis has indicated that 2R,3R-dihydromyricetin (DMY), the flavonoid compound isolated from pine needles of *Cedrus deodara* (Roxb.) Loud (family: Pinaceae), can inhibit the expression of proteins related to the attachment and accumulation stages of *Staphylococcus aureus* biofilm formation and downregulate key enzymes involved in cell wall formation [12]. Via transcriptomic analysis, baicalin has been demonstrated to disrupt several biological pathways related to antibiotic resistance, biofilm formation, cell membrane permeability, and bacterial virulence [13]. This progress has enriched the current understanding of the antibacterial effects of small molecules.

Due to the increasing risk of antibiotic resistance in bacteria to small molecules, linear and short antimicrobial peptides have been extensively investigated in recent years [14,15]. Although many linear and short antimicrobial peptides have been identified [16,17,18,19,20], transcriptomic analysis currently remains highly limited due to the fact that many of these linear and short antimicrobial peptides can rapidly disrupt bacterial cell membranes, inhibit bacterial growth or kill bacteria over the course of only several minutes [16,21,22]. Similar to linear and short antimicrobial peptides, defensins are broadly defined as cysteine-rich antimicrobial peptides, and their antibacterial functions have also been widely studied in recent years [6,8,23]. Due to their cysteine-stabilized spatial structures, many defensins have been observed to inhibit bacterial growth considerably more slowly than linear and short antimicrobial peptides [21,24,25,26]. Therefore, the broad-spectrum antimicrobial activity of defensins represents a unique opportunity for the development of new antibacterial drugs. Given the current widespread application of omics-based analyses in various fields, the highly limited global information concerning defensin effects against different bacteria that is obtained from omics-based analyses can undoubtedly influence the research and development of defensins as potential drugs.

In our previous study, a representative scorpion defensin BmKDfsin4 with two distinct hydrophobic and basic residue clusters among a total of 37 amino acid residues was extensively investigated, and a BLAST online (https://www.ncbi.nlm.nih.gov/) search of the protein database revealed approximately 100 similar defensin sequences (>70%), and the two hydrophobic and basic residue clusters play different roles during the inhibition of bacterial growth [27]. Unlike most linear and short antimicrobial peptides that rapidly disrupt bacterial cell membranes over the course of several minutes and may be more cytotoxic, BmKDfsin4 can moderately inhibit bacterial growth by inhibiting cell wall synthesis and promoting bacterial autolysis rather than by disrupting bacterial cell membranes [27]. In this study, transcriptomic and proteomic analyses were adopted to explore and identify global information regarding the defensin BmKDfsin4 and its effects on *Staphylococcus aureus*. In the presence of the defensin BmKDfsin4, many differentially expressed genes (DEGs) and differentially expressed proteins (DEPs) are involved in various aspects of bacterial physiological activities. Further analysis revealed that BmKDfsin4 significantly upregulated the expression of ribosome and protein synthesis genes in *Staphylococcus aureus*, which aligned well with the substantial downregulation of amino acid synthesis. Additionally, the bacterial cell wall synthesis pathway was significantly downregulated by BmKDfsin4, whereas the synthesis pathways of key cell wall components (such as peptidoglycan and teichoic acid) were notably suppressed. Bacterial metabolism inhibition by BmKDfsin4 was also a key factor driving the gradual death of the bacteria. These transcriptomic and proteomic findings increase the knowledge of the antimicrobial mechanisms of defensins, which could accelerate the research and development of defensins as novel antibiotics.

## 2. Results

### 2.1. Bactericidal Effect of BmKDfsin4 on S. aureus AB94004

**Figure 1:**
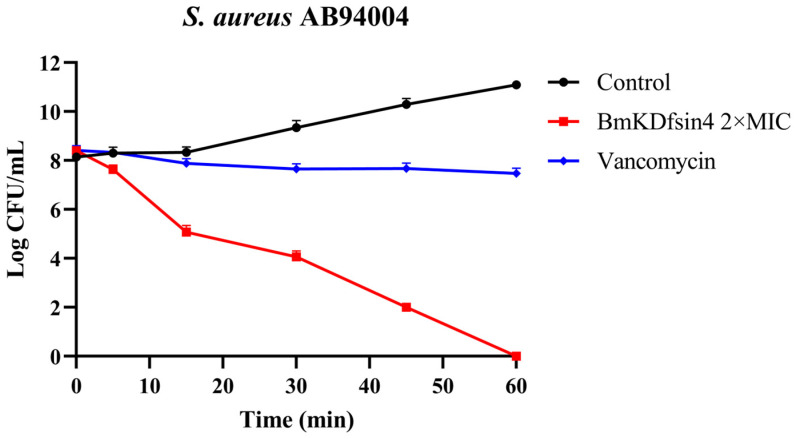
The killing kinetics of 2 × MIC BmKDfsin4 against *S. aureus* AB94004. The positive control was vancomycin at a concentration of 2 µg/mL, and the final concentration of BmKDfsin4 was 2 × MIC. *n* ≥ 3.

To assess the bactericidal activity of BmKDfsin4 against *Staphylococcus aureus*, a bactericidal kinetic assay of the representative strain *S. aureus* AB94004 was performed. The results demonstrated that at 2 × MIC, BmKDfsin4 exhibited a moderate bactericidal effect on *S. aureus* AB94004, thereby resulting in bacterial eradication within 1 h. Notably, the bactericidal rate of BmKDfsin4 was faster than that of the positive control (vancomycin) (Figure 1). Similarly, bacterial growth was observed to be effectively inhibited by BmKDfsin4 within 6 h in our previous study [27]. These results demonstrate that BmKDfsin4 not only effectively suppresses the proliferation of *S. aureus* but also exerts a moderate bactericidal effect.

### 2.2. Global Transcriptomic and Proteomic Analyses of BmKDfsin4-Treated S. aureus AB94004

**Figure 2:**
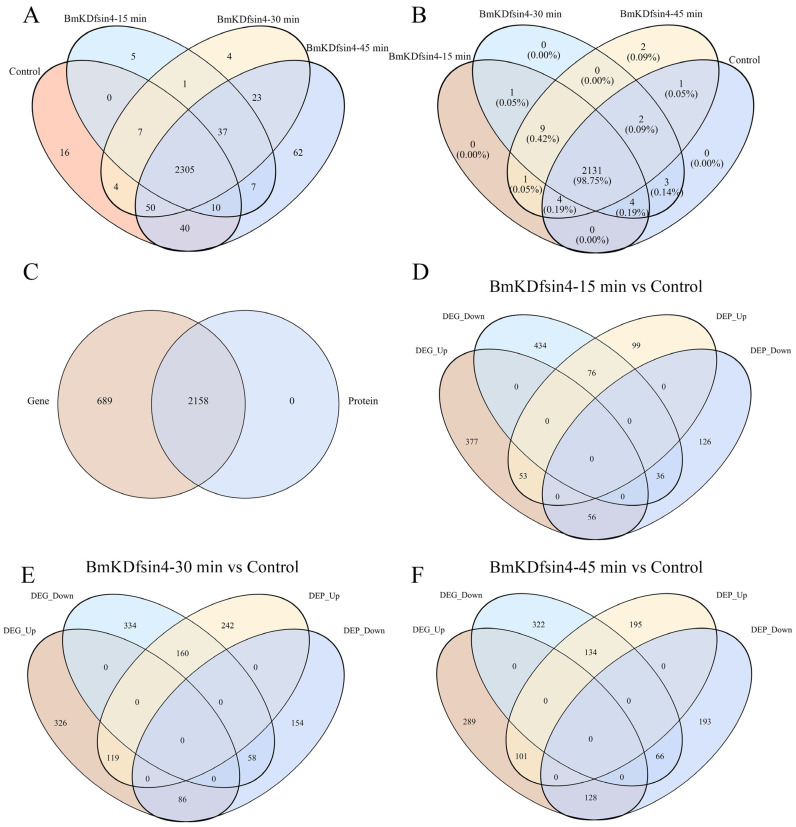
The expression levels of genes or proteins in the different groups: (**A**) Comparison of the number of expressed genes between the different groups; (**B**) comparison of the number of expressed proteins between the different groups; (**C**) proteins or genes that were common and unique to the proteome or transcriptome, respectively; (**D**–**F**) correlation between upregulated or downregulated genes and proteins after treatment with BmKDfsin4 for 15, 30, and 45 min, respectively.

We performed transcriptomic and proteomic analyses on *S. aureus* AB94004 treated with BmKDfsin4 for 15, 30, and 45 min and subsequently characterized the differentially expressed genes (DEGs) and differentially expressed proteins (DEPs) before and after defensin treatment. Gene expression analysis revealed that the treatment of *S. aureus* AB94004 with BmKDfsin4 induced the expression of several genes that were not expressed in the control group. Compared with the 2432 genes expressed in the control group, 50 and 65 other genes were specifically expressed at 15 min and 30 min, respectively (Figure 2A). The number of specifically expressed genes increased to 127 after 45 min of BmKDfsin4 treatment (Figure 2A). Although the comparison of the proteomic and transcriptomic results revealed that all of the 2158 detected protein-encoding genes were also present in the transcriptomic data, 689 transcripts were not detected at the protein level (Figure 2C). Similarly, after treatment with BmKDfsin4 for 15, 30, and 45 min, 11, 10, and 12 proteins, respectively, were specifically expressed (Figure 2B). The observed disparities in gene and protein expression underscored the profound impact of BmKDfsin4 treatment on *S. aureus* AB94004, which is a phenomenon that became enhanced with prolonged exposure. An analysis of these specifically expressed genes and proteins may reveal crucial elements that are pivotal to identifying defensins or identifying the effects of defensins on bacterial growth.

**Figure 3:**
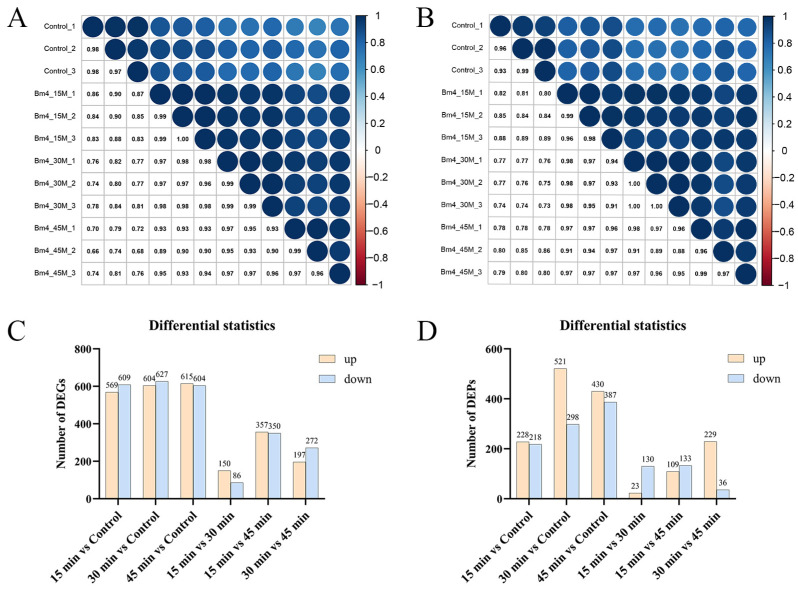
Intergroup correlation analysis and statistical analysis of differentially expressed genes or proteins: (**A**) Transcriptomic intergroup and intragroup correlation analyses; (**B**) proteomic intergroup and intragroup correlation analyses; (**C**) statistical analysis of differentially expressed genes (DEGs) between the groups; (**D**) statistical analysis of differentially expressed proteins (DEPs) between the groups. Yellow and blue indicate upregulated genes and downregulated genes, respectively.

Based on the transcriptomic results, intergroup and intragroup correlation analyses revealed that the gene expression of *S. aureus* AB94004 treated with BmKDfsin4 for 15, 30, or 45 min was significantly different from that of the control group (Figure 3A). The DEG analysis revealed that, compared with no treatment, treatment with BmKDfsin4 for 15, 30, and 45 min resulted in 569, 604, and 615 genes that were significantly downregulated, respectively, as well as 609, 627, and 604 genes that were significantly upregulated, respectively (Figure 3C). Among these genes, the most significant difference in gene expression was observed between the 15 min and 45 min treatment groups, wherein 357 genes were upregulated and 350 genes were downregulated (Figure 3C). Similarly, proteomic analysis revealed that the protein expression in BmKDfsin4-treated *S. aureus* AB94004 significantly differed from that in the control group (Figure 3B). The DEP analysis revealed that treatment with BmKDfsin4 led to significant downregulation of 228, 521, and 430 proteins at 15, 30, and 45 min, respectively. Conversely, 218, 298, and 387 proteins were significantly upregulated at these corresponding time points (Figure 3D). Analyses of DEGs and DEPs at different treatment time points demonstrated both positive and negative correlations between gene and protein expression. For example, after 30 min of treatment with BmKDfsin4, 119 genes were significantly upregulated, and the proteins encoded by these genes were also significantly upregulated. Similarly, 58 genes were significantly downregulated in the 30 min-treated groups, and the proteins encoded by these genes were also significantly downregulated. Conversely, 86 genes were upregulated after 30 min of BmKDfsin4 treatment; however, the corresponding proteins encoded by these genes were downregulated. Similarly, 160 genes in the 30 min-treated group were downregulated; however, the corresponding proteins encoded by these genes were significantly upregulated (Figure 2E). This phenomenon was also demonstrated in the treatment assays at both 15 and 45 min (Figure 2D,F).

To respond to BmKDfsin4, *S. aureus* AB94004 clearly exhibited remarkable alterations in both gene and protein expression. These changes were related not only to the direct consequences of defensin influence on bacteria but also to the adaptive responses of bacteria to defend against defensin.

### 2.3. Functional Enrichment Analysis of DEGs and DEPs

**Figure 4:**
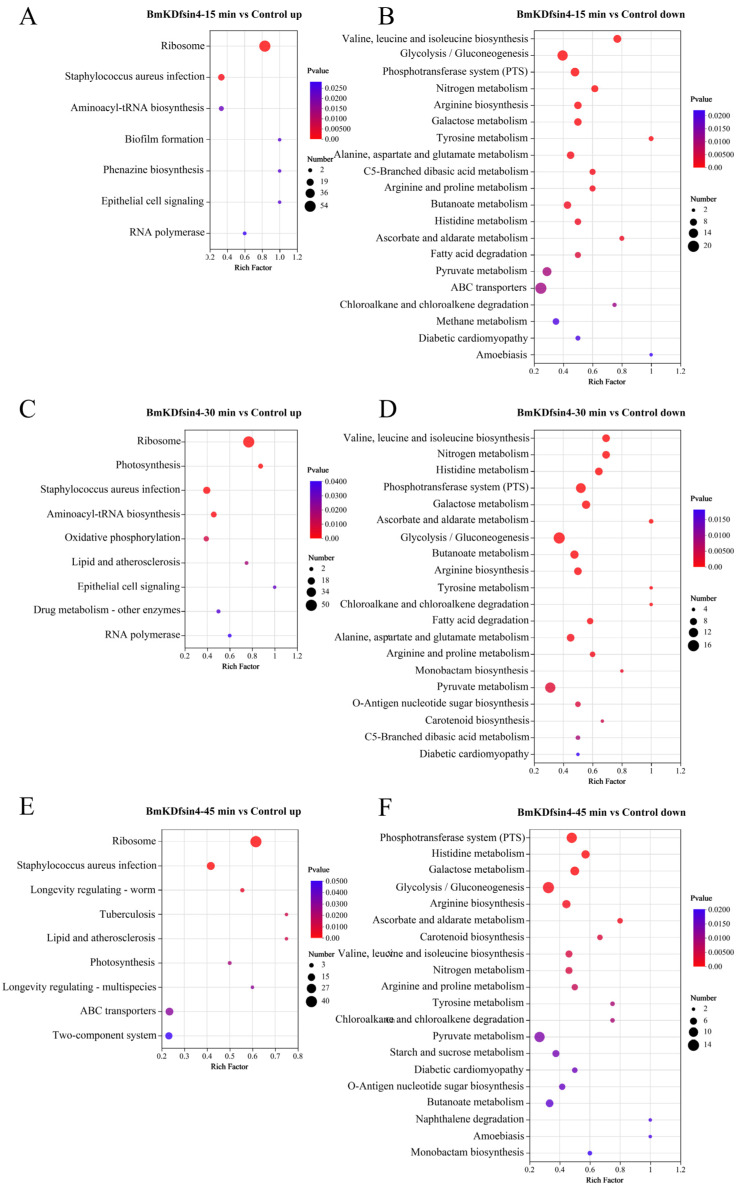
KEGG pathway enrichment analysis of DEGs: (**A**,**C**,**E**) representative KEGG pathways enriched by the upregulated genes in *S. aureus* AB94004 treated with BmKDfsin4 for 15, 30, and 45 min, respectively; (**B**,**D**,**F**) representative top 20 KEGG pathways enriched by the downregulated genes in *S. aureus* AB94004 treated with BmKDfsin4 for 15, 30, and 45 min, respectively.

A large number of DEGs and DEPs were identified to be helpful for understanding the influence of the scorpion defensin known as BmKDfsin4 on the physiological functions of *S. aureus*, including its biological processes (such as cell proliferation and cell wall synthesis) (Figure 2 and Figure 3), which could be determined via functional enrichment analysis based on classical KEGG pathway enrichment analysis and Gene Ontology (GO) functional enrichment analysis. By using KEGG pathway enrichment analysis of the genes exhibiting expression levels that were upregulated in *S. aureus* AB94004 following treatment with BmKDfsin4 for 15, 30, and 45 min, a subset of pathways were significantly upregulated in the treated bacteria (Figure 4A,C,E). Notably, after the treatment (regardless of whether treatment occurred for a short duration of 15 min or a longer duration of 45 min), the significant upregulation of the bacterial ribosome pathway was consistently observed (Figure 4A,C,E), which strongly suggested a substantial impact on protein synthesis-related processes within the bacteria. After pathway enrichment analysis of upregulated genes in the bacteria after 15 and 30 min of treatment, significant upregulation in the biosynthesis pathway of aminoacyl-tRNA was identified (Figure 4A,C), which is a crucial process involving the transfer of amino acids to the ribosome and their integration into the elongating polypeptide chain, thereby contributing to the addition of amino acids and affecting protein synthesis within the bacterial cell. Further analysis of the significantly downregulated DEGs revealed that the KEGG pathways predominantly involved amino acid synthesis and metabolism, thereby encompassing processes such as valine, leucine, and isoleucine biosynthesis; arginine biosynthesis; tyrosine metabolism; and alanine, aspartate, and glutamate metabolism (Figure 4B,D,F). Moreover, several enriched metabolic pathways, such as nitrogen metabolism, galactose metabolism, and glycolysis pathways, were also significantly downregulated. These crucial metabolic processes were notably suppressed in *S. aureus* AB94004 following treatment with BmKDfsin4, thus indicating a substantial impact of BmKDfsin4 on the metabolic pathways of the bacteria.

**Figure 5:**
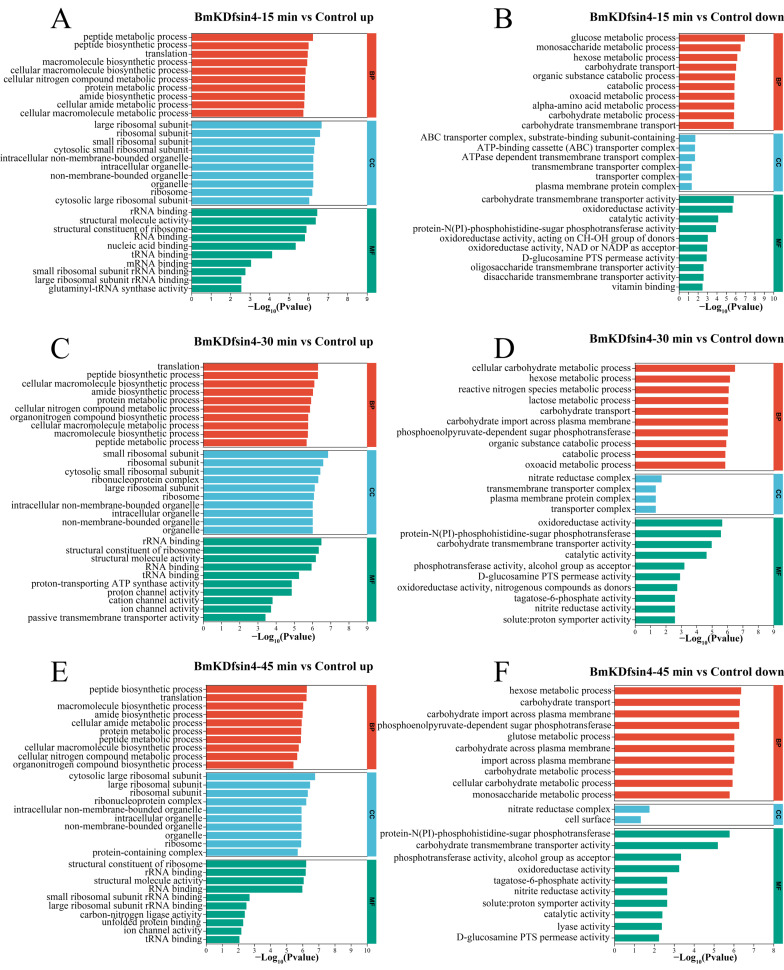
GO enrichment analysis of DEGs: (**A**,**C**,**E**) representative GO enrichment analyses of the upregulated genes in *S. aureus* AB94004 treated with BmKDfsin4 for 15, 30 and 45 min, respectively; (**B**,**D**,**F**) representative GO enrichment analyses of the downregulated genes in *S. aureus* AB94004 treated with BmKDfsin4 for 15, 30 and 45 min, respectively. The top 10 biological processes (BPs), cell component (CC) and molecular function (MF) terms were selected according to the *p* value (*p* < 0.05).

GO functional analysis was also used to determine the bacterial changes in biological process (BP), cellular component (CC), and molecular function (MF) terms relative to those in the control group after treatment with BmKDfsin4 for different durations. The upregulated genes associated with biological processes were observed to be mainly associated with the synthesis and metabolism of biomacromolecules, such as peptide synthesis and metabolism processes, translation processes, protein metabolism processes, and the biosynthesis of cellular nitrogen compounds (Figure 5A,C,E). These biological processes were significantly upregulated as early as 15 min of treatment (Figure 5A) and remained highly upregulated at 30 and 45 min of treatment (Figure 5C,E). The results of the upregulation analysis of cellular components indicated a particularly significant upregulation of ribosome-related components, including large ribosomal subunits, small ribosomal subunits, and ribosomal protein components, which are essential constituents of ribosomes (Figure 5B,D,F). Clearly, the upregulation of ribosome-related components may be associated with protein synthesis. Furthermore, some organelle components within the bacteria were also upregulated, such as intracellular organelles and intracellular nonmembrane-bound organelles, which indicated that the normal physiological activities of *S. aureus* after treatment with BmKDfsin4 were significantly affected (Figure 5B,D,F). Similarly, GO functional analysis of the downregulated DEGs revealed significant upregulation in BP, CC, and MF. Unlike the functions enriched by the upregulated DEGs, these downregulated genes are related to the metabolism of *S. aureus* AB94004, such as glucose metabolism; monosaccharide and hexose metabolism; and carbohydrate transport and metabolism processes (Figure 5B,D,F). Among the downregulated genes observed at 15 min and 30 min, those genes involved in the metabolic processes of oxoacid were enriched (Figure 5B,D). The genes that were downregulated between 30 and 45 min were enriched in the phosphoenolpyruvate-dependent sugar phosphotransferase system, which is a pivotal system involved in sugar metabolism; specifically, the suppression of this system can lead to disruptions in bacterial sugar metabolism, thereby potentially influencing bacterial survival intricacies (Figure 5D,F). In the enrichment of molecular functions among the downregulated genes, significant downregulation was predominantly demonstrated for transport complexes, including transmembrane and ATP-binding cassette (ABC) transport complexes (Figure 5B), which may be related to energy production and utilization in bacteria [28,29,30,31]. In terms of molecular function, significant downregulation was mainly associated with catalytic reactions within bacteria, such as the activities of oxidoreductases, phosphoserine transaminases, hydrolases, and nitrate reductases (Figure 5B,D,F). The downregulation of these enzyme activities resulted in the disruption of normal biochemical reactions within the bacteria.

KEGG pathway enrichment analysis and GO functional enrichment analysis of the DEPs were further conducted to investigate the impact of BmKDfsin4 on *S. aureus*, which could reflect the changes that occurred at the bacterial protein level. The KEGG pathway enrichment analysis revealed that several crucial pathways, such as ribosome-related pathways, were significantly upregulated (Table 1), which is highly consistent with the transcriptomic results (Figure 4). In addition, pathways such as riboflavin metabolism, the pentose phosphate pathway, cysteine and methionine metabolism, nitrogen metabolism, the biosynthesis of cofactors, and purine metabolism were significantly upregulated, thus indicating that BmKDfsin4 could significantly impact the metabolism of *S. aureus* AB94004 (Table 1). Conversely, several downregulated pathways were still observed, including pathways related to benzoate metabolism, RNA polymerase, phospholipid biosynthesis, and PTS (Table 1). Teichoic acid is an essential component of the cell wall of *S. aureus*, and the downregulation of the teichoic acid biosynthesis pathway indicated a potential hindrance of bacterial cell wall synthesis, which could impact bacterial proliferation.

**Figure 6:**
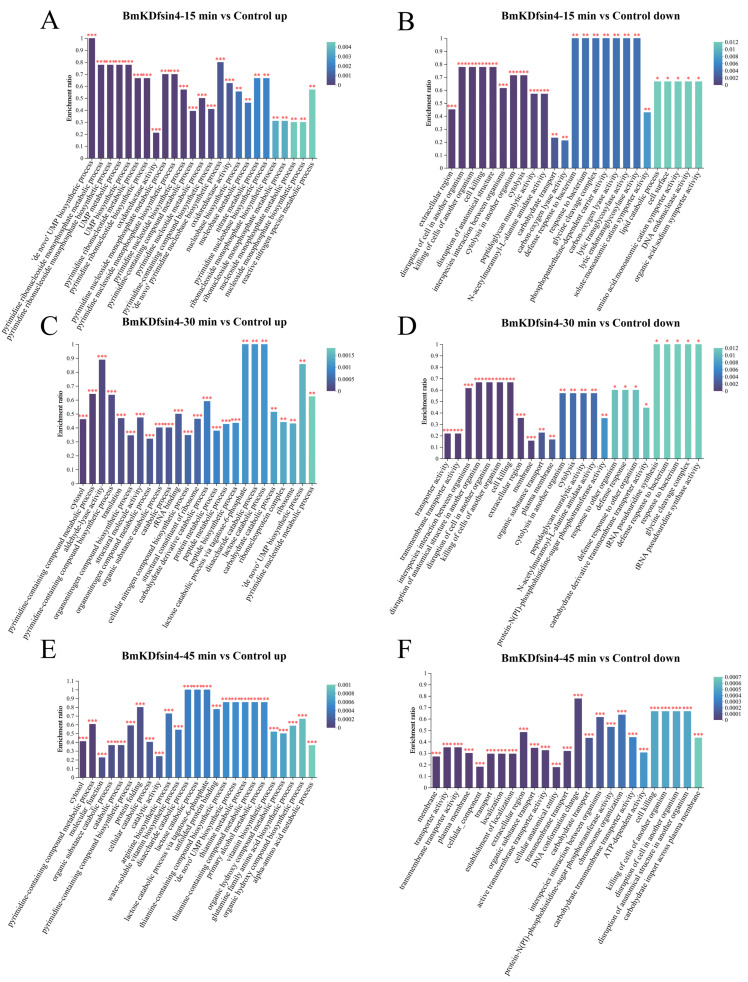
GO enrichment analysis of DEPs: (**A**,**C**,**E**) representative top 20 GO terms enriched by the upregulated proteins in *S. aureus* AB94004 treated with BmKDfsin4 for 15, 30, and 45 min, respectively; (**B**,**D**,**F**) representative top 20 GO terms enriched by the downregulated proteins in *S. aureus* AB94004 treated with BmKDfsin4 for 15, 30 and 45 min, respectively. *, *p* < 0.05; **, *p* < 0.01; ***, *p* < 0.001.

During the GO enrichment analysis of the upregulated genes in the context of differential expression, various biological processes that are crucially linked to the synthesis and metabolism of essential substances vital for life activities were enriched. In the GO analysis of the upregulated DEPs in *S. aureus* AB94004 treated with BmKDfsin4 at different time points, several processes were identified, such as pyrimidine ribonucleotide biosynthesis and metabolism; UMP synthesis and metabolism; the ‘de novo’ pyrimidine nucleobase biosynthetic process; and translation processes (Figure 6A,C,E). However, the enriched downregulated GO terms were related to specific catalytic activities, such as peptidoglycan muralytic activity, carbon–oxygen lyase activity, lytic transglycosylase activity, ATP-dependent activity, and N-acetylmuramoyl-L-alanine amidase activity (Figure 6B,D,F). In addition, there were also downregulated GO terms observed that were related to cellular components and molecular functions, such as the extracellular region, glycine cleavage complex, transporter activity, transmembrane transporter activity, and peptidoglycan muralytic activity (Figure 6B,D,F).

In summary, an abundance of pathways within the bacteria treated with BmKDfsin4 was demonstrated to be either upregulated or downregulated via KEGG pathway enrichment analysis and GO functional enrichment analysis of differentially expressed genes and proteins. These pathways identified via KEGG pathway analysis are crucial for bacterial survival and encompass functions such as proliferation and metabolism. Similarly, the GO analysis revealed significant alterations in biological processes, cellular components, and molecular functions that are vital for bacterial survival. Undoubtedly, these upregulated or downregulated changes were unfavorable for bacterial viability.

### 2.4. Effects of BmKDfsin4 on the Ribosome Assembly and Protein Synthesis of S. aureus

The ribosome particle in bacteria, which is comprised of a 30S small subunit (containing 16S rRNA and 21 proteins) and a 50S large subunit (containing 5S and 23S rRNA, along with 31 proteins), plays an extremely important role in the survival of bacteria; specifically, mRNAs bind to ribosomes to undergo translation to produce proteins.

**Figure 7:**
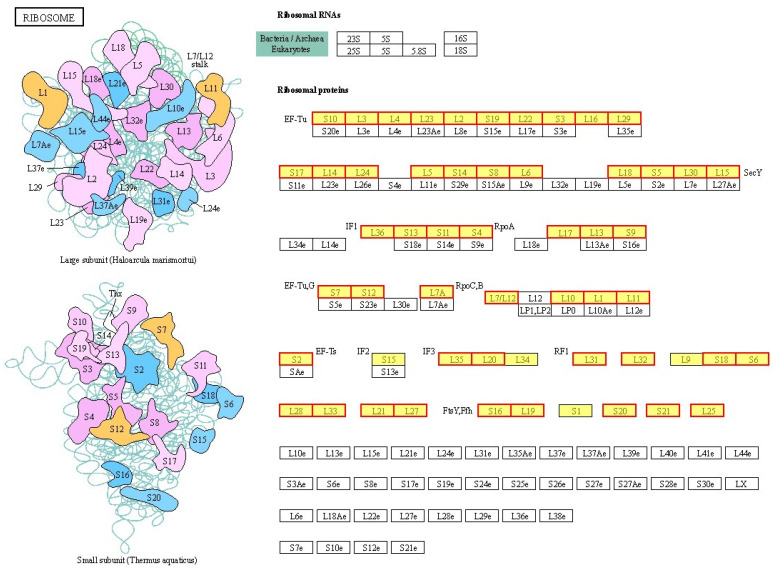
Pathways of ribosomes enriched through DEGs. The ribosome pathways obtained from the KEGG pathway enrichment analysis of differentially expressed genes in *S. aureus* treated with BmKDfsin4. The yellow rectangles represent the enriched genes or proteins, whereas the red borders indicate significant upregulation. *p* value < 0.05. The pathway figure was adapted from a public database (KEGG PATHWAY: map03010).

After treatment with BmKDfsin4, there was a significant increase observed in the expression levels of genes encoding components of the ribosome in *S. aureus* AB94004, both in the 50S and 30S subunits, as well as in ribosomal proteins such as L3, L4, and L16 (Figure 7 and Table S1) [32]. KEGG pathway enrichment analysis of DEPs in *S. aureus* AB94004 treated with BmKDfsin4 revealed that the ribosome pathway was significantly upregulated in bacteria treated for 15, 30, or 45 min (Figure 4). This pathway included a total of 58 genes (Table S1), most of which were significantly upregulated after defensin treatment (Figure 7) [32]. Similarly, the GO functional analysis of the DEGs revealed that the ribosomal components in the cellular component category were significantly enriched among the enriched GO terms. These components included the large ribosomal subunit, small ribosomal subunit, cytosolic large ribosomal subunit, and ribonucleoprotein complex, which are key components of the bacterial ribosome (Figure 5). KEGG pathway enrichment analysis of the DEPs revealed significant upregulation of the ribosome pathway following BmKDfsin4 treatment (Table 1). These findings closely aligned with the results of the KEGG enrichment analysis of the DEGs, thereby suggesting that the ribosome was consistently upregulated at both the gene and protein levels. In the GO functional analysis of the DEPs, 26 ribosome-related proteins associated with the BP category and 1 protein associated with the CC category were significantly upregulated (Table 2). In *S. aureus* AB94004 treated with BmKDfsin4, the significant upregulation of ribosomal expression at both the gene and protein levels demonstrated enhanced ribosome production.

Ribosomes are essential for binding mRNAs and translating them into proteins. Although these proteins often require further processing to attain their specific functions, the critical role of ribosomes cannot be ignored. In this study, the observed upregulation of ribosomes likely indicated an accelerated rate of protein synthesis. The KEGG pathway enrichment analysis of the DEGs revealed that the aminoacyl-tRNA biosynthesis pathway was significantly upregulated in *S. aureus* treated with BmKDfsin4 (Figure 4). BmKDfsin4 treatment significantly increased the expression levels of enzymes associated with tRNA amino acid transport in *S. aureus*, such as proline–tRNA ligase (*proS*), serine–tRNA ligase (*serS*), and histidine–tRNA ligase (*hisS*) (Table 3), which are closely related to protein expression. The upregulated GO terms were enriched in biological processes such as translation, peptide biosynthesis, and macromolecule biosynthesis in the GO analysis of the DEGs. Similarly, molecular functions such as rRNA binding, tRNA binding, and mRNA binding were also significantly enriched (Figure 5). Additionally, significant upregulation was observed in processes such as translation, peptide synthesis, and protein folding in the GO functional enrichment analysis of the DEPs (Figure 6). We also observed that the protein level of translation initiation factor IF-1 (*infA*), which is involved in regulating the efficiency and fidelity of the formation of translation–initiation complexes, was significantly increased by BmKDfsin4 exposure. Interestingly, the level of the ribosome-associated translation inhibitor (*raiA*), which can interact with the 70S and 30S ribosome subunits and suppress translation initiation in bacteria, was significantly reduced by BmKDfsin4.

All of these findings suggest that BmKDfsin4 profoundly impacts *Staphylococcus aureus*, whereby it significantly alters protein synthesis by upregulating ribosome expression and related tRNA aminoacylation processes, as bacteria have attempted to counteract this effect.

### 2.5. Reduction in Amino Acid Biosynthesis and Metabolism in S. aureus Due to BmKDfsin4 Treatment

**Figure 8:**
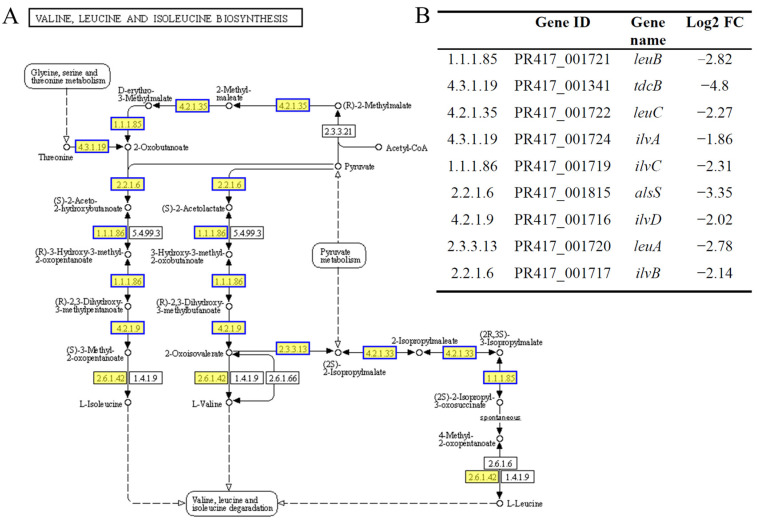
Pathways of valine, leucine, and isoleucine biosynthesis. (**A**) The valine, leucine, and isoleucine biosynthesis pathways were obtained from the KEGG pathway enrichment analysis of DEGs in *S. aureus* treated with BmKDfsin4. The yellow rectangles represent the enriched genes or proteins, whereas the blue borders indicate significant downregulation (*p* < 0.05); (**B**) the detailed expression of DEGs related to the valine, leucine, and isoleucine biosynthesis pathways. The pathway figure was adapted from a public database (KEGG PATHWAY: map00290).

Amino acids are essential raw materials for the synthesis of proteins and nucleotides, and their proper synthesis and metabolism are crucial for the survival of *S. aureus*. In our study, BmKDfsin4 treatment significantly reduced the biosynthesis and metabolism of various amino acids in *S. aureus*, as clearly demonstrated by the KEGG pathway enrichment analysis of the DEGs. The valine, leucine, and isoleucine biosynthesis pathways were significantly downregulated at 15, 30, and 45 min after BmKDfsin4 treatment (Figure 4). Some key genes in these pathways, such as *leuB*, *leuC*, *tdcB*, *ilvA*, and *ilvB* (which play critical roles in the biosynthesis of amino acids in bacteria), were significantly downregulated (Figure 8) [33]. Similarly, amino acid metabolism pathways, including the metabolism pathways of arginine, proline, and tyrosine, as well as metabolism pathways of alanine, asparagine, and glutamine, were markedly suppressed (Figure 4 and Figure 8).

Additionally, carbamoyl-phosphate synthetase and glutamine synthetase (GlnA) are crucial enzymes that catalyze the conversion of ammonia into organic nitrogen. GlnA catalyzes the formation of L-glutamine from L-glutamate, and both molecules are important intermediates for the synthesis of tryptophan, histidine, and other amino acids. Notably, the expression of the *glnA* gene, which encodes GlnA, was significantly decreased after BmKDfsin4 treatment, and the corresponding protein was not detected at the protein level (Table 4). Other genes related to amino acid synthesis and metabolism, such as *ald*, *puuE*, and *metX*, were also significantly downregulated at both the gene and protein levels, which further indicated that BmKDfsin4 treatment led to a significant decrease in both the biosynthesis and metabolism of amino acids (Table 4). The *argJ* gene, which is involved in arginine synthesis, exhibited increased expression at the gene level. However, the protein encoded by *argJ* (ArgJ) was significantly downregulated, with a log_2_ fold change (FC) value of −16.61 being observed; *argH* and *argG* were also downregulated, which suggested that arginine synthesis was hindered by BmKDfsin4 treatment (Table 4).

The metabolism of aspartate and glutamine provides the carbon and nitrogen backbone for purine rings, thus indicating that these metabolism pathways are closely associated with purine biosynthesis. The genes encoding aspartate aminotransferase family proteins, glutamate synthase, Glu/Leu/Phe/Val dehydrogenase, and type I glutamate–ammonia ligases were significantly downregulated after BmKDfsin4 treatment (Table 4). The gene *purA*, which encodes L-glutamate gamma-semialdehyde dehydrogenase (an enzyme crucial for the metabolism and synthesis of glutamate), was also significantly downregulated after treatment with BmKDfsin4. These enzymes are closely involved in the synthesis and metabolism of aspartate and glutamate. In the KEGG pathway enrichment analysis of the DEGs, the metabolic pathways associated with these amino acids were also downregulated (Figure S1). Amidophosphoribosyl transferase (PurF) and phosphoribosylglycinamide formyltransferase (PurN) are involved in the biosynthesis of hypoxanthine and adenine nucleotides, and the genes encoding these enzymes were also significantly downregulated after BmKDfsin4 treatment (Table 4). Therefore, BmKDfsin4 treatment significantly reduced the synthesis and metabolism of amino acids, which could be lethal to *S. aureus* and pose a serious threat to its survival.

### 2.6. Inhibition of Cell Wall Biosynthesis in S. aureus AB94004 Due to BmKDfsin4 Treatment

The cell wall of *S. aureus* is primarily composed of peptidoglycan and teichoic acids, which help to maintain bacterial shape and provide protection to bacteria. During bacterial proliferation, the continuous synthesis of new cell wall components is essential, thereby demonstrating that proper cell wall biosynthesis is one of the key requirements for bacterial survival and growth. In our previous study, after *S. aureus* was treated with BmKDfsin4 at a 2 × MIC concentration, a decrease in cell wall thickness was observed as the treatment time increased. Additionally, the expression of genes related to cell wall synthesis (such as *murG*) was downregulated [27].

**Figure 9:**
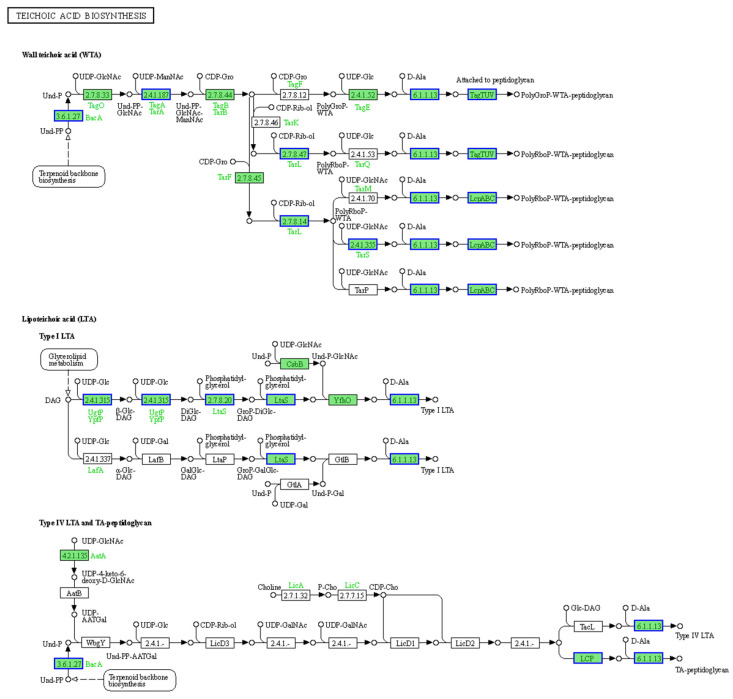
Pathway of *S. aureus* teichoic acid biosynthesis. The teichoic acid biosynthesis pathway obtained from the KEGG pathway enrichment analysis of DEPs in *S. aureus* treated with BmKDfsin4. The green rectangles represent the enriched genes or proteins, whereas the blue borders indicate significant downregulation (*p* value < 0.05). The pathway figure was adapted from a public database (KEGG PATHWAY: map00552).

In this study, the synthesis pathway of teichoic acid, which is a key component of *S. aureus* AB94004, was significantly downregulated according to the KEGG pathway enrichment analysis of DEPs. The key proteins involved in this biosynthesis process, such as the teichoic acid biosynthesis protein TagA, peptidoglycan teichoic acid transferase, and UDP-N-acetylmuramoyl-L-alanyl-D-glutamate synthetase (MurD), were significantly downregulated after treatment with BmKDfsin4 (Figure 9, Table 5). The synthesis of peptidoglycan, which is another key component of the *S. aureus* cell wall, was impaired. LCP family proteins, which are crucial for peptidoglycan synthesis, were significantly downregulated due to BmKDfsin4 treatment (Figure 9, Table 5). Furthermore, the analyses of the DEGs and DEPs revealed that penicillin-binding protein (PBP), which is involved in the final step of bacterial cell wall synthesis, was significantly downregulated at both the gene and protein levels. These results indicated that *S. aureus* could not form a complete cell wall, thereby leading to significant inhibition of its proliferation (Table 5), which aligned well with previous findings that BmKDfsin4 inhibited the cell wall biosynthesis of *S. aureus* AB94004 and further suppressed bacterial growth [27].

### 2.7. Inhibitory Effect of BmKDfsin4 on the Metabolism of S. aureus AB9004

Normal metabolic activity is a critical condition for the survival of *S. aureus.* Metabolic disruption (whether elicited via accelerated or diminished metabolic processes) can be lethal to bacteria. In this study, BmKDfsin4 treatment significantly reduced the metabolism of *S. aureus* AB94004. In the KEGG pathway enrichment analysis of the DEGs, several pathways, such as nitrogen metabolism, the phosphotransferase system, galactose metabolism, glycolysis/gluconeogenesis, and fatty acid degradation, were significantly downregulated (Figure 4). These metabolic processes are crucial for the overall survival and function of *S. aureus*. For example, glycolysis is the process by which glucose is broken down to generate ATP, whereas gluconeogenesis is the reverse process that generates glucose from noncarbohydrate precursors. These pathways are vital for energy production and maintaining cellular functions, particularly under different nutrient conditions. The significant downregulation of these pathways suggested that BmKDfsin4 impaired the ability of bacteria to efficiently perform essential metabolic processes, which could ultimately inhibit their growth and survival. And the GO functional enrichment analysis of the downregulated DEGs revealed significant enrichment in processes such as glucose, monosaccharide, hexose, and other carbohydrate metabolism; reactive nitrogen species metabolism; and oxoacid metabolism (Figure 5). Carbohydrates are a primary energy source for bacteria. Disruption of the metabolism of glucose and other carbohydrates can impair the ability of bacteria to generate ATP and other essential metabolic intermediates, thus leading to reduced growth and energy production. This scenario could hinder the ability of bacteria to thrive in environments where carbohydrates are the main nutrient source. Overall, the downregulation of these key metabolic pathways suggested that BmKDfsin4 severely impaired the ability of bacteria to produce energy, manage stress, and synthesize essential cellular components, which could hinder their growth, survival, and proliferative abilities.

The activities of enzymes in *S. aureus* directly influence bacterial metabolism. Enzymes are crucial for catalyzing metabolic reactions, and their activities determine the efficiency and rate of various biochemical processes, including nutrient uptake, energy production, and the synthesis of essential cellular components. After the bacteria were treated with BmKDfsin4, the activities of several key enzymes were significantly reduced. For example, the activities of enzymes such as oxidoreductases, protein-N(PI)-phosphohistidine-sugar phosphotransferase, phosphotransferases, and tagatose-6-phosphate kinase were significantly downregulated according to the GO analysis of differentially expressed genes (Figure 5). These enzymes play important roles in various metabolic processes, and their reduced activities can disrupt the ability of bacteria to perform essential biochemical reactions, which may impair bacterial growth and survival. These results indicated that BmKDfsin4 treatment significantly impaired bacterial metabolism by downregulating key enzymes and metabolic pathways, thereby disrupting energy production, nutrient processing, and essential biosynthesis processes.

## 3. Discussion

Currently, the increasing risk of antibiotic-resistant bacteria has accelerated extensive investigations of the antibacterial functions and mechanisms of small molecules, linear antimicrobial peptides, and cysteine-rich defensins in recent years [4,5,6,8]. The global differential expression profiles of bacterial genes and proteins can enhance the functional knowledge of antibacterial small molecules based on transcriptomic and proteomic analyses [9,10], which suggests that antibiotics typically have a single and specific antimicrobial mechanism [34,35]. The broad-spectrum antimicrobial activities of various defensins have demonstrated a unique opportunity for the development of new antibacterial drugs [6,8,36]; however, the highly limited global information concerning defensin effects against different bacteria from omics-based analyses can potentially influence the research and development of defensins as potential drugs.

Scorpions are considered to be living fossils that maintain an ancient anatomy, and their defensins likely play important survival roles in different environments. Several similar defensins were observed in the scorpion *Mesobuthus martensii*, and BmKDfsin4 was easily synthesized via a recombinant strategy [27]. BmKDfsin4, a 37-amino acid residue defensin from *Mesobuthus martensii*, inhibits the growth of Gram-positive bacteria [27]. A large number of defensins from other species (such as ticks, etc.) are highly similar to BmKDfsin4 and exhibit similar antibacterial activity [27]. In this study, transcriptomic and proteomic analyses were adopted to explore the effects of the defensin BmKDfsin4 on *S. aureus*, which provided global information concerning the effects of defensins against bacteria at both the gene and protein levels.

The moderate bactericidal effect of BmKDfsin4 is related to its cysteine-stabilized spatial structure and two distinct hydrophobic and basic residue clusters [27], which are different from most linear and short antimicrobial peptides that rapidly disrupt bacterial cell membranes over the course of several minutes [21,26]. Previous RT-qPCR results have revealed that the expression of genes associated with bacterial proliferation is significantly altered [27], and transcriptomic analysis also revealed changes in the expression of these genes (Figure S2). Moreover, this unique structural feature of BmKDfsin4 also resulted in relatively low cytotoxicity toward certain mammalian cells [37]. Subsequent transcriptomic and proteomic analyses revealed that over 1000 genes and nearly 500 proteins in *S. aureus* were significantly differentially regulated after BmKDfsin4 treatment (Figure 2 and Figure 3). Further analysis of the upregulated and downregulated genes and proteins from the KEGG pathway and GO functional enrichment analyses provided valuable insights into the antibacterial effects of BmKDfsin4. The ribosome-related pathway was the most significantly upregulated pathway at different time points following BmKDfsin4 treatment (Figure 4 and Figure 7). Among the 58 ribosome-associated genes that are involved in this pathway, the majority of the genes were significantly upregulated (Table S1). The aminoacyl-tRNA biosynthesis pathway and genes related to enzymes associated with tRNA amino acid transport were also significantly upregulated (Figure 4). Similarly, the ribosome pathway was prominently enriched and exhibited significant upregulation of the expression of numerous ribosomal proteins according to the KEGG pathway enrichment analysis of the DEPs (Table 1 and Table 2). Moreover, several biological processes were significantly enriched in the upregulated GO terms, including translation; ribosomal subunit formation (including both large and small ribosomal subunits); and the binding of rRNA, mRNA, and tRNA (Figure 5). After exposure to BmKDfsin4, the protein level of the translation initiation factor IF-1 (*infA*), which regulates the efficiency and fidelity of translation initiation complex formation, was significantly increased, whereas the expression of the ribosome-associated translation inhibitor (*raiA*) was significantly suppressed. The upregulation of these pathways (along with the associated genes and proteins) suggested alterations in the translation process, which may accelerate protein synthesis. This scenario could represent a survival strategy employed by the bacteria in response to BmKDfsin4 exposure. Notably, this novel feature contrasts with the antimicrobial effects of small molecules such as tannic acid, which is a plant-derived compound that inhibits ribosome assembly and downregulates IF-1 while also upregulating *raiA*, thereby suppressing protein synthesis in *S. aureus* [38]. Similarly, an aromatic alcohol compound has also been observed to inhibit ribosome assembly and disrupt protein synthesis in *Escherichia coli* [39]. In contrast, exposure to BmKDfsin4 could accelerate protein synthesis in *S. aureus* AB94004, which may initially seem to be advantageous for bacterial survival; however, due to the fact that increased protein synthesis can accelerate energy consumption, a potential metabolic burden may ultimately be exerted on the bacteria.

Although BmKDfsin4 accelerated protein synthesis in *S. aureus*, several pathways related to amino acid biosynthesis and metabolism, including the valine, leucine, isoleucine, and arginine biosynthesis pathways, as well as the threonine, arginine, proline, and histidine metabolism pathways, were significantly downregulated after BmKDfsin4 treatment (Figure 4). Key genes such as *leuB*, *leuC*, *tdcB*, *ilvA*, *ilvB*, *ald*, *puuE*, and *metX* were also significantly downregulated (Figure 8 and Table 4). In addition, the *glnA* gene, which encodes carbamoylphosphate synthetase and glutamine synthetase [40], as well as other enzymes involved in the synthesis of intermediates essential for the biosynthesis of tryptophan, histidine, and other amino acids, were also significantly downregulated (Table 4). These changes indicated a significant restriction in amino acid synthesis and metabolism, which may critically affect bacterial survival and metabolic activities. The downregulation of amino acid synthesis and metabolism further affected protein synthesis, which seemed to be contradictory to the previously mentioned acceleration of protein synthesis. However, when bacteria lack sufficient amino acids for protein synthesis, the increased expression of ribosomal proteins and tRNAs may alleviate the crisis involving reduced protein synthesis caused by amino acid shortages. Asparagine and glutamine play crucial roles in providing the carbon backbone for purine ring synthesis [38]. Following treatment with BmKDfsin4, the metabolic pathways of asparagine and glutamine in *S. aureus* AB94004 were significantly downregulated (Figure S1). Additionally, the genes encoding key enzymes (including aspartate aminotransferase family proteins, glutamate synthase, Glu/Leu/Phe/Val dehydrogenase, and type I glutamate–ammonia ligase) were also significantly downregulated (Table 4). These findings suggested that the inhibition of purine synthesis could disrupt a range of essential metabolic processes in bacteria.

Peptidoglycan and teichoic acids are essential components of the cell wall in Gram-positive bacteria. After treatment with BmKDfsin4, the synthesis of these two substances was significantly inhibited in *S. aureus* (Figure 9 and Table 5). The teichoic acid biosynthesis pathway was enriched in the downregulated genes, and the proteins related to this pathway were observed to be significantly downregulated in the proteomic analysis, including LCP family proteins and penicillin-binding protein (PBP) (Table 5); transcriptomics revealed significant changes in the expression of key genes involved in bacterial cell wall synthesis (Figure S2), which aligned well with our previous experimental observations of the significant downregulation of PBP protein-related genes [27]. Bacteria need to continuously synthesize new cell walls during proliferation; however, BmKDfsin4 significantly inhibits the synthesis of new cell walls, which correspondingly indirectly suppresses bacterial proliferation [27]. More importantly, BmKDfsin4 treatment significantly suppressed the metabolism of *S. aureus*. Several metabolic pathways (such as carbohydrate metabolism and glycolysis) were notably downregulated, as revealed by pathway enrichment analysis (Figure 4). The activities of several key enzymes involved in bacterial metabolism (such as oxidoreductases) were significantly downregulated according to the GO functional enrichment analysis (Figure 5 and Figure 6). This reduction in metabolic activity suggested that ATP production in *S. aureus* was inhibited. When considering that the accelerated protein synthesis process results in a considerable amount of energy being consumed, this combination of different effects is likely lethal to bacteria, thereby severely compromising their survival. In addition, the transcriptomic results revealed that the expression of bacterial autolysis-related genes (such as *atlE* and *arlR/S*) was upregulated, which was consistent with our previous results (Figure S2) [27], thus suggesting that BmKDfsin4 can indeed promote bacterial autolysis.

In summary, transcriptomic and proteomic analyses revealed the multiple mechanisms of BmKDfsin4 action against *S. aureus*; specifically, BmKDfsin4 accelerated bacterial protein synthesis while simultaneously inhibiting bacterial metabolism, thereby leading to a significant reduction in ATP production. Accelerated protein synthesis further increased energy consumption, which correspondingly accelerated bacterial cell death. Additionally, BmKDfsin4 inhibited bacterial proliferation by suppressing cell wall synthesis and downregulating amino acid synthesis and metabolism, which further accelerated the progression of bacterial death.

Currently, small-molecule drugs used clinically to treat bacterial infections typically inhibit bacterial proliferation through a single mechanism. This study comprehensively analyzed the multi-target antimicrobial mechanisms of BmKDfsin4 from transcriptomic and proteomic perspectives. Further differential expression analyses provided a theoretical foundation for developing clinical antimicrobial agents, offering new potential directions for treating drug-resistant bacterial infections. These novel findings clearly provide in-depth insights into the molecular mechanism of the defensin BmKDfsin4 against *S. aureus*, and these multiple antibacterial mechanisms could reduce the emergence of resistant bacteria in the development of new antibacterial agents in the future.

## 4. Materials and Methods

### 4.1. Bactericidal Kinetics

Firstly, scorpion defensin BmKDfsin4 is obtained by prokaryotic expression, as detailed in our previous work [27]. Next, *Staphylococcus aureus* AB94004 was cultured in 5 mL of Luria-Bertani medium at 37 °C with continuous shaking at 200 rpm until it reached the mid-logarithmic phase. One milliliter was then transferred to a tube, and BmKDfsin4 was added to achieve a final concentration of 2 × MIC (0.5 μM) [27]. The culture was incubated at 37 °C and continuous shaking at 200 rpm, and samples of 10 µL were collected at 0, 5, 15, 30, 45, and 60 min. These samples were serially diluted and plated on antibiotic-free agar plates, which were incubated overnight at 37 °C in an inverted position for colony counting. Vancomycin treatment at 2 μg/mL was used as a positive control, and this concentration effectively inhibited the growth of *S. aureus*.

### 4.2. RNA Extraction

The *S. aureus* AB94004 was cultured in liquid Luria-Bertani medium until it reached the logarithmic growth phase, with an OD_630_ of approximately 0.3 being observed. At this point, the cells were treated with scorpion defensin BmKDfsin4 at a final concentration of 2 × MIC and incubated for 15, 30, or 45 min, after which the harvested cells were rapidly frozen in liquid nitrogen and subsequently ground. Total RNA was extracted from the tissue via the CTAB method. Briefly, the CTAB extraction buffer was prepared by adding 2% β-mercaptoethanol (Sinopharm Chemical Reagent Co., Ltd., Shanghai, China) and preheating the buffer to 65 °C. *S. aureus* was resuspended in the buffer and incubated at 65 °C for 5 min. An equal volume of chloroform–isoamyl alcohol (Sinopharm Chemical Reagent Co., LTD, Shanghai, China) (24:1) was added, followed by vigorous shaking and centrifugation at 12,000 rpm for 10 min at 4 °C. The supernatant was collected, and the extraction was repeated. To precipitate the RNA, an equal volume of 4 M lithium chloride was added, and the mixture was incubated at 4 °C for 2 h. The RNA was subsequently pelleted via centrifugation at 12,000 rpm for 10 min at 4 °C, washed with ethanol to remove impurities, air-dried, dissolved in DEPC-treated water, and stored at −80 °C.

### 4.3. Transcriptomic Analysis

The transcriptomic analysis was performed by Shanghai Majorbio Bio-pharm Technology Co., Ltd. (Shanghai, China), and the relevant analysis steps are briefly described below. Ribosomal RNA (rRNA) depletion instead of poly(A) purification was performed by RiboCop rRNA Depletion Kit for Mixed Bacterial Samples (lexogen, Greenland, NH, USA), and then all mRNAs were broken into short (200 bp) fragments by adding fragmentation buffer firstly. Secondly, double-stranded cDNA was synthesized using random hexamer primers. When the second strand cDNA was synthesized, dUTP was incorporated in place of dTTP. Then, the synthesized cDNA was subjected to end-repair, phosphorylation, and ‘A’ base addition according to Illumina’s library construction protocol. RNA-seq transcriptome library was prepared following Illumina^®^ Stranded mRNA Prep, Ligation (San Diego, CA, USA) using total RNA. The paired-end RNA-seq library was sequenced with the Illumina Novaseq 6000 (Illumina Inc., San Diego, CA, USA). The processing of original images to sequences, base-calling, and quality value calculations. The clean reads were obtained by removing low-quality sequences, reads with more than 10% of N bases (unknown bases), and reads containing adaptor sequences. Gene expression analysis was performed using the RSEM tool (RNA-Seq gene expression estimation with read mapping uncertainty), and differential expression analysis was performed using the DESeq2 tool (Bioconductor - DESeq2).

### 4.4. Protein Extraction and Proteomic Analysis

BmKDfsin4 was added to *S. aureus* in the logarithmic growth phase to achieve a final concentration of 2 × MIC (0.5 μM). The samples were collected by centrifugation at the following time points: 0, 15, 30, and 45 min. The bacteria were resuspended in a 1 mL lysis buffer (8 M urea containing protease inhibitors), incubated on ice, and subjected to sonication for 2 min. The mixture was then incubated on ice for an additional 30 min for cell lysis. Afterward, the samples were centrifuged at 12,000 rpm at 4 °C to collect the supernatant.

Proteomic analysis was also performed by Shanghai Majorbio Bio-pharm Technology Co., Ltd. (Shanghai, China). Briefly, the total protein concentration of the extracted samples was determined using the bicinchoninic acid assay (BCA). For digestion, 100 µg of the protein sample was mixed with trypsin (enzyme/protein = 1:50) and incubated overnight at 37 °C. After digestion, the protein samples were desalted and subsequently analyzed by liquid chromatography–tandem mass spectrometry (VanquishNeo-Astral, Thermo, Waltham, MA, USA) using the data-independent acquisition (DIA) mode for data collection. The raw DIA data were analyzed using Spectronaut™ 18/DIA-NN (Thermo Fisher Scientific, Massachusetts, USA)to obtain quantitative results. Afterward, the quantitative results were subjected to differential expression analysis, followed by Gene Ontology (GO) functional enrichment analysis and Kyoto Encyclopedia of Genes and Genomes (KEGG) pathway enrichment analysis.

### 4.5. Bioinformatics and Statistical Analysis

Differentially expressed genes (DEGs) and differentially expressed proteins (DEPs) were subjected to Gene Ontology (GO) functional enrichment analysis and Kyoto Encyclopedia of Genes and Genomes (KEGG) pathway enrichment analysis using the GO database (Gene Ontology Resource) and the KEGG database (KEGG: Kyoto Encyclopedia of Genes and Genomes), respectively. The data were analyzed via the online Majorbio Cloud Platform (www.majorbio.com). Genes and proteins with *p* values < 0.05 and |fold change| ≥ 1.5 (|log_2_FC| ≥ 0.58) were considered to be differentially expressed.

## Figures and Tables

**Table 1:** KEGG pathway enrichment analysis of DEPs in *S. aureus* AB94004 treated with BmKDfsin4.

Pathway ID	KEGG Description	*p* Value	Rich Factor	Regulation
map00740	Riboflavin metabolism	5.42 × 10^−6^	0.90	up
map00240	Pyrimidine metabolism	2.39 × 10^−5^	0.42	up
map03010	Ribosome	3.20 × 10^−5^	0.48	up
map00030	Pentose phosphate pathway	1.69 × 10^−4^	0.62	up
map00270	Cysteine and methionine metabolism	5.95 × 10^−4^	0.57	up
map01240	Biosynthesis of cofactor	7.59 × 10^−4^	0.37	up
map00910	Nitrogen metabolism	1.14 × 10^−3^	0.46	up
map00230	Purine metabolism	2.65 × 10^−3^	0.43	up
map01232	Nucleotide metabolism	2.85 × 10^−3^	0.47	up
map01240	Biosynthesis of cofactor	5.21 × 10^−3^	0.31	up
map00730	Thiamine metabolism	7.60 × 10^−3^	0.60	up
map00360	Phenylalanine metabolism	8.93 × 10^−3^	1.00	up
map01502	Vancomycin resistance	1.93 × 10^−2^	0.67	up
map00983	Drug metabolism—other enzyme	2.06 × 10^−2^	0.63	up
map00350	Tyrosine metabolism	3.02 × 10^−2^	0.75	up
map00750	Vitamin B6 metabolism	3.02 × 10^−2^	0.75	up
map00362	Benzoate degradation	1.20 × 10^−2^	0.50	down
map03020	RNA polymerase	2.90 × 10^−2^	0.60	down
map00552	Teichoic acid biosynthesis	3.40 × 10^−2^	0.41	down
map02060	Phosphotransferase system (PTS)	3.40 × 10^−2^	0.41	down

**Table 2:** Proteins related to the ribosome of *S. aureus* AB94004 treated with BmKDfsin4.

Accession	Protein Name	Log_2_ FC	*p* Value	Regulation	GO Term
PR417_000186	Ribosomal_S14	0.68	0.004935	up	BP
PR417_000260	Ribosomal_S15	3.86	0.01018	up	BP
PR417_000310	Ribosomal_L28	0.98	0.001746	up	BP
PR417_000560	Ribosomal_S20p	1.21	0.0006025	up	BP
PR417_000570	Ribosomal_S21	2.01	0.004936	up	BP
PR417_001047	Ribosomal_S10	0.88	0.0007706	up	BP
PR417_001051	Ribosomal_L2	1.53	0.0000644	up	BP
PR417_001052	Ribosomal_S19	2.92	0.0007064	up	BP
PR417_001054	Ribosomal_S3_C	0.70	0.0002152	up	BP
PR417_001055	Ribosomal_L16	1.29	0.002452	up	BP
PR417_001057	Ribosomal_S17	1.51	0.001027	up	BP
PR417_001059	Ribosomal_L24	2.01	0.001125	up	BP
PR417_001064	Ribosomal_L18p	1.59	0.0003322	up	BP
PR417_001066	Ribosomal_L30	1.65	0.0000517	up	BP
PR417_001067	Ribosomal_L27A	0.77	0.000431	up	BP
PR417_001073	Ribosomal_S11	0.84	0.0000539	up	BP
PR417_001217	Ribosomal_S4	0.75	0.001019	up	BP
PR417_001258	Ribosomal_L35p	3.92	0.0004422	up	BP
PR417_001259	Ribosomal_L20	0.77	0.002422	up	BP
PR417_001303	Ribosomal_S1	0.72	0.001404	up	CC
PR417_001445	Ribosomal_L31	1.21	0.006626	up	BP
PR417_001541	Ribosomal_S18	1.93	0.0001338	up	BP
PR417_001543	Ribosomal_S6	2.54	0.0000811	up	BP
PR417_001894	Ribosomal_L9	0.70	0.006551	up	BP
PR417_002081	Ribosomal_L10	0.67	0.0001408	up	BP
PR417_002082	Ribosomal_L12	1.68	0.0007194	up	BP
PR417_002088	Ribosomal_S7	0.94	0.003338	up	BP

**Table 3:** Genes related to aminoacyl–tRNA biosynthesis in *S. aureus* treated with BmKDfsin4.

Gene ID	Gene Name	Gene Description	Log_2_ FC	*p* Value	Regulation
PR417_000270	*proS*	proline–tRNA ligase	1.05	1.01 × 10^−34^	up
PR417_000515	*hisS*	histidine–tRNA ligase	2.00	9.38 × 10^−57^	up
PR417_000530	*alaS*	alanine–tRNA ligase	0.95	4.72 × 10^−16^	up
PR417_000580	*glyS1*	glycine–tRNA ligase	1.22	3.33 × 10^−46^	up
PR417_001207	*tyrS*	tyrosine–tRNA ligase	0.94	1.14 × 10^−6^	up
PR417_001255	*thrS*	threonine–tRNA ligase	1.03	1.54 × 10^−9^	up
PR417_001275	*valS*	valine–tRNA ligase	0.81	2.02 × 10^−17^	up
PR417_001324	*asnS*	asparagine–tRNA ligase	0.87	3.27 × 10^−14^	up
PR417_001745	*leuS*	leucine–tRNA ligase	1.18	5.81 × 10^−26^	up
PR417_001888	*serS*	serine–tRNA ligase	0.60	4.58 × 10^−8^	up
PR417_002019	*lysS*	lysine–tRNA ligase	0.63	4.96 × 10^−11^	up
PR417_002406	*ileS*	isoleucine–tRNA ligase	1.85	9.62 × 10^−77^	up
PR417_001117	*pheT*	phenylalanine–tRNA ligase subunit beta	1.39	2.01 × 10^−26^	up
PR417_001118	*pheS*	phenylalanine–tRNA ligase subunit alpha	1.05	9.27 × 10^−17^	up

**Table 4:** Genes and proteins related to amino acid biosynthesis and metabolism.

Accession	Gene Name	Protein ID	Description	Log_2_ FC	
				Gene	Protein
PR417_001228	*ald*	HDK3247889.1	Alanine dehydrogenase	−0.55	−0.72
PR417_000076	*puuE*	HDK3246759.1	Aspartate aminotransferase family protein	−1.91	−0.67
PR417_001891	*metX*	HDK3248527.1	Homoserine O-acetyltransferase	−0.07	−0.60
PR417_001358	*argJ*	HDK3248015.1	Bifunctional glutamate N-acetyltransferase/amino-acid acetyltransferase ArgJ	0.86	−16.61
PR417_000873	*argH*	HDK3247540.1	Argininosuccinate lyase	−3.66	
PR417_000874	*argG*	HDK3247541.1	Argininosuccinate synthase	−2.77	
PR417_001186	*purF*	HDK3247847.1	Amidophosphoribosyl transferase	−0.53	-
PR417_001184	*purN*	HDK3247845.1	Phosphoribosyl glycinamide formyl transferase	−0.41	-
PR417_000028	*pruA*	HDK3246711.1	L-glutamate gamma-semialdehyde dehydrogenase	−0.90	-
PR417_000076	-	HDK3246759.1	Aspartate aminotransferase family protein	−1.91	−0.668
PR417_002017	*gltB*	HDK3248649.1	Glutamate synthase large subunit	−1.45	
PR417_002018	*gltD*	HDK3248650.1	Glutamate synthase subunit beta	−1.23	
PR417_000871	-	HDK3247538.1	Glu/Leu/Phe/Val dehydrogenase	−1.08	
PR417_000225	*glnA*	HDK3246901.1	Type I glutamate–ammonia ligase	−0.96	-

**Table 5:** Proteins related to cell wall biosynthesis in *S. aureus*.

Accession	Protein Name	Protein ID	Description	Log_2_ FC
PR417_000847	DltC	HDK3247514.1	D-alanine-activating enzyme/D-alanine-D-alanyl	−0.84
PR417_000449	LtaS	HDK3247125.1	Polyglycerol-phosphate lipoteichoic acid synthase LtaS	−0.95
PR417_002396	MurD	HDK3249008.1	UDP-N-acetylmuramoyl-L-alanyl-D-glutamate synthetase	−0.59
PR417_000273	UppS	HDK3246949.1	UDP pyrophosphate synthase UppS	−0.85
PR417_001798	-	HDK3248439.1	LCP protein family	−1.00
PR417_000593	PBP	HDK3247268.1	Penicillin-binding protein	−0.60
PR417_002630	-	HDK3249238.1	Polyisoprenyl-teichoic acid–peptidoglycan teichoic acid transferase	−0.62
PR417_000365	TagA	HDK3247041.1	Teichoic acid biosynthesis protein TagA	−0.74

## Data Availability

The original contributions presented in this study are included in the article/Supplementary Material. Further inquiries can be directed to the corresponding author(s).

## Supplementary Materials

The following supporting information can be downloaded at: https://www.mdpi.com/article/10.3390/molecules30071542/s1, Figure S1: The metabolism pathway of alanine, aspartate and glutamate; Figure S2: Expression levels of genes related to bacterial proliferation, cell wall synthesis and autolysis; Table S1: The DEGs of ribosome of *S. aureus* treated by BmKDfsin4 with different time.
