# Supplementary material for: Insights into the Antimicrobial Mechanisms of a Scorpion Defensin on Staphylococcus aureus Using Transcriptomic and Proteomic Analyses
Source: Molecules. 2025 Mar 30;30(7):1542. doi: 10.3390/molecules30071542 (PMC11990343; doi:10.3390/molecules30071542)
Supplement: Supplementary file 1 [file molecules-30-01542-s001.zip › molecules-3540241-supplementary.pdf]

Table S1. The DEGs of ribosome of S. aureus treated by BmKDfsin4 with different time

Gene_ID	Gene name	Gene description	Log2 FC		
			15 min VS Control	30 min VS Control	45 min VS Control
PR417_000187	*rpmG*	50S ribosomal protein L33	2.38	2.30	1.22
PR417_000260	*rpsO*	30S ribosomal protein S15	1.55	0.94	-0.45
PR417_000278	*rpsB*	30S ribosomal protein S2	2.17	2.05	1.44
PR417_000293	*rplS*	50S ribosomal protein L19	2.51	2.05	0.74
PR417_000296	*rpsP*	30S ribosomal protein S16	2.87	2.27	1.24
PR417_000310	-	50S ribosomal protein L28	1.40	1.55	1.16
PR417_000499	*rplU*	50S ribosomal protein L21	2.17	1.72	1.01
PR417_000500	-	ribosomal-processing cysteine protease Prp	2.30	1.93	1.53
PR417_000501	*rpmA*	50S ribosomal protein L27	1.46	1.11	0.02
PR417_000560	-	30S ribosomal protein S20	2.31	1.98	1.09
PR417_000567	*prmA*	50S ribosomal protein L11 methyltransferase	1.21	1.14	1.90
PR417_000570	-	30S ribosomal protein S21	1.52	1.13	0.30
PR417_000594	*rpmG*	50S ribosomal protein L33	1.89	1.67	1.12
PR417_001047	*rpsJ*	30S ribosomal protein S10	2.72	2.50	1.56
PR417_001048	*rplC*	50S ribosomal protein L3	2.53	2.27	1.94
PR417_001049	*rplD*	50S ribosomal protein L4	2.55	2.35	1.99
PR417_001050	*rplW*	50S ribosomal protein L23	2.99	2.65	2.07
PR417_001051	*rplB*	50S ribosomal protein L2	2.42	2.11	1.39
PR417_001052	*rpsS*	30S ribosomal protein S19	2.77	2.29	2.09
PR417_001053	*rplV*	50S ribosomal protein L22	2.77	2.34	1.97
PR417_001054	*rpsC*	30S ribosomal protein S3	2.44	2.13	1.79
PR417_001055	*rplP*	50S ribosomal protein L16	2.51	2.18	1.74
PR417_001056	*rpmC*	50S ribosomal protein L29	2.55	2.42	1.45
PR417_001057	*rpsQ*	30S ribosomal protein S17	2.37	1.99	1.58
PR417_001058	*rplN*	50S ribosomal protein L14	1.97	1.59	1.16
PR417_001059	*rplX*	50S ribosomal protein L24	2.69	2.30	2.17
PR417_001060	*rplE*	50S ribosomal protein L5	2.37	2.05	1.66
PR417_001061	-	type Z 30S ribosomal protein S14	2.57	2.17	1.75
PR417_001062	*rpsH*	30S ribosomal protein S8	2.55	2.23	1.88
PR417_001063	*rplF*	50S ribosomal protein L6	2.45	2.11	1.86
PR417_001064	*rplR*	50S ribosomal protein L18	2.29	2.02	1.77
PR417_001065	*rpsE*	30S ribosomal protein S5	2.15	1.95	1.43
PR417_001066	*rpmD*	50S ribosomal protein L30	1.74	1.50	1.22
PR417_001067	*rplO*	50S ribosomal protein L15	1.73	1.53	1.18
PR417_001071	*rpmJ*	50S ribosomal protein L36	1.81	1.47	1.49
PR417_001072	*rpsM*	30S ribosomal protein S13	1.46	1.15	0.82
PR417_001073	*rpsK*	30S ribosomal protein S11	1.63	1.43	1.29
PR417_001075	*rplQ*	50S ribosomal protein L17	1.47	1.26	0.52
PR417_001080	*rplM*	50S ribosomal protein L13	1.35	1.13	0.66
PR417_001081	*rpsI*	30S ribosomal protein S9	1.24	0.85	-0.05

PR417_001129	*rpmF*	50S ribosomal protein L32	1.43	0.96	-0.58
PR417_001217	*rpsD*	30S ribosomal protein S4	2.36	1.74	0.67
PR417_001258	*rpmI*	50S ribosomal protein L35	2.83	2.10	1.25
PR417_001259	*rplT*	50S ribosomal protein L20	2.02	1.29	0.35
PR417_001445	-	type B 50S ribosomal protein L31	2.49	2.27	1.02
PR417_001541	*rpsR*	30S ribosomal protein S18	2.99	2.41	1.11
PR417_001543	-	30S ribosomal protein S6	2.86	2.56	2.24
PR417_001879	*rpmH*	50S ribosomal protein L34	1.30	0.39	-0.80
PR417_002036	-	50S ribosomal protein L25	2.30	2.00	1.10
PR417_002076	*rpmG*	50S ribosomal protein L33	0.64	0.20	-1.17
PR417_002079	*rplK*	50S ribosomal protein L11	2.28	2.12	1.42
PR417_002080	*rplA*	50S ribosomal protein L1	2.62	2.38	1.06
PR417_002081	-	50S ribosomal protein L10	2.67	2.18	1.14
PR417_002082	*rplL*	50S ribosomal protein L7/L12	2.80	2.23	1.00
PR417_002086	-	ribosomal L7Ae/L30e/S12e/Gadd45 family protein	2.23	2.35	0.93
PR417_002087	*rpsL*	30S ribosomal protein S12	1.96	1.94	1.09
PR417_002088	*rpsG*	30S ribosomal protein S7	1.80	1.74	0.73

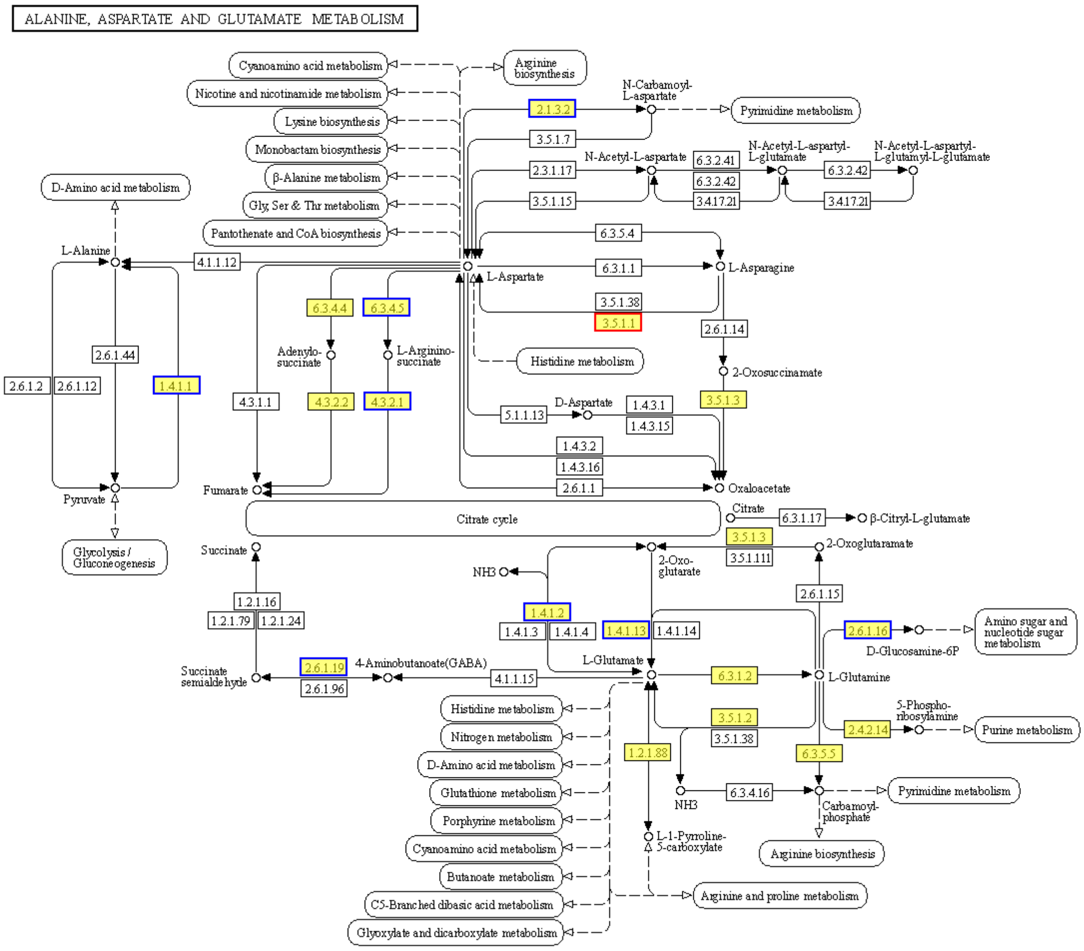

Figure S1. The metabolism pathway of alanine, aspartate and glutamate.

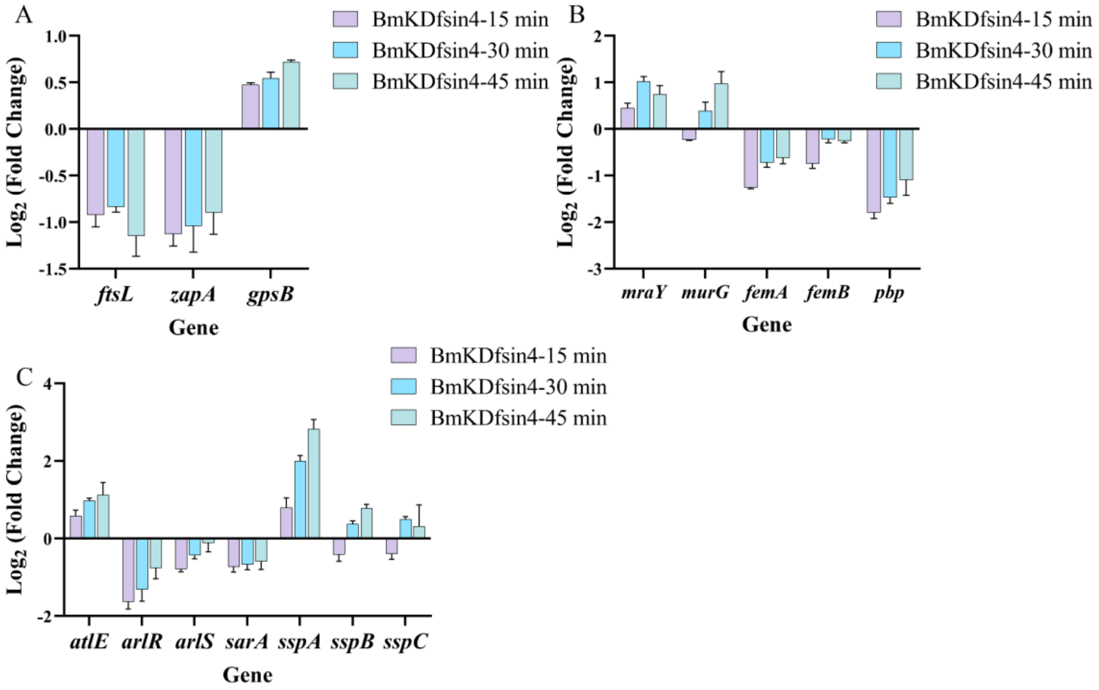

Figure S2. Expression levels of genes related to bacterial proliferation, cell wall synthesis and autolysis. A) Expression levels of genes related to bacterial proliferation; B) Expression levels of genes related to cell wall synthesis; C) Expression levels of genes related to autolysis. $n \geq 3$.
